# A CD21 low phenotype, with no evidence of autoantibodies to complement proteins, is consistent with a poor prognosis in CLL

**DOI:** 10.18632/oncotarget.5404

**Published:** 2015-10-03

**Authors:** Eva-Maria Nichols, Rachel Jones, Rachael Watson, Chris J. Pepper, Chris Fegan, Kevin J. Marchbank

**Affiliations:** ^1^ Institute of Cellular Medicine, Newcastle University, Newcastle-upon-Tyne, UK; ^2^ Institute of Cancer & Genetics, Cardiff University School of Medicine, Cardiff, UK

**Keywords:** CLL, poor prognosis, CD21, complement, B cell

## Abstract

B-cell chronic lymphocytic leukemia (CLL) is characterized by differential BCR signaling and autoimmune complications. Complement modulates B-cell function via C3d and CD21 cross-linked to the B-cell receptor (BCR). We hypothesized that CD21 contributes to BCR signaling and participates in the autoimmunity associated with CLL. We analyzed CD21 expression on 106 CLL patient samples and matched serum from 50 patients for the presence of soluble CD21 and autoantibodies to CR2, CR1, MCP and FH. CD21 expression on CLL B-cells was significantly lower than that expressed on B-cells from age-matched controls (*P* < 0.0001) and was inversely correlated with soluble CD21 (*r*^2^ = −0.41). We found no evidence of autoantibody to any complement regulator. Low CD21 expression correlated to prognostic subsets of CLL patients, i.e. cases with unmutated *IGHV* genes (*P* = 0.0006), high CD38 (*P* = 0.02) and high ZAP70 expression (*P* = 0.0017). Low CD21 expression was inversely correlated to the levels of phosphotyrosine induced in CLL cells following BCR ligation with αIgM (*r*^2^=–0.21). Importantly, lower CD21 expression was also predictive for reduced overall survival (*P* = 0.005; HR = 2.7). In conclusion, we showed that reduced expression of CD21 on CLL B-cells appears functionally relevant and was associated with poor clinical outcomes.

## INTRODUCTION

Chronic lymphocytic leukemia (CLL) is a clinically heterogeneous disease and characterized by the clonal expansion of functionally incompetent B-cells in the lymph node, bone marrow and blood. Previous studies have shown two types of CLL based on their *IGHV* mutation status and it is now thought that unmutated CLL is derived from unmutated mature CD5+ B-cells whereas mutated CLL is derived from a distinct, CD5+CD27+, post-germinal center B-cell subset [1–3]. CLL cells display an activated B1 and regulatory B-cell phenotype [4, 5]; they are considered antigen experienced, possibly following recognition of self-antigen, with a very restricted BCR repertoire [6, 7]. CLL is characterized by constitutive activation of BCR signaling pathways but with variable responsiveness to antigen ligation; associated with co-expression of CD38 via ZAP70 [8–10]. It is widely accepted that BCR signaling leads to survival signals and resistance against anergy [11, 12]. The recent finding that BTK inhibitors (acting downstream of the BCR) can kill CLL cells has highlighted the important role of the BCR in the pathogenesis of CLL [13–15]. For a significant minority of patients autoimmunity is a clinical problem due to auto-immune hemolytic anemia, immune thrombocytopenia purpura and low immunoglobulins [16, 17].

CD21 participates in the BCR co-receptor complex (CD21, CD19 and CD81). Co-ligation of CD21 and the BCR by C3dg-opsonised antigen can result in a thousand-fold reduction of the B-cell activation threshold [18–20] and is sufficient to protect B-cells from FAS-mediated apoptosis [21]. Natural ligands of CD21 include the C3 activation fragments iC3b, C3dg and C3d [22]. CD21 plays an important role in the selection for high-affinity B-cells as well as the development and maintenance of B-cell memory [22]. While the BCR co-receptor function of CD21 predominates, CD21 also mediates effects independent of the BCR including the induction of the transcription factor NF-κB, the production of interleukin-6 (IL-6) and the internalization of antigen [23, 24].

C3d, a key ligand for CD21, is generated through activation of the complement system via the alternative, classical or lectin pathway. This involves generation of C3 convertases followed by rapid control by complement regulators, such as CD46, CD55 and CD35 [25, 26]. Thus, any alteration of complement activation can result in increased ligand availability for CD21 and/or other cell bound complement regulators, which may result in increased B-cell signaling. Recent studies have shown that Rituximab, used to treat CLL, partially kills through complement-mediated mechanisms and indeed some CLL patients have reduced serum complement levels causing Rituximab resistance [27, 28].

CLL is characterized by constitutive BCR activation and subsequent NF-κB signaling, albeit with variable responsiveness of the BCR to antigen ligation [29]. Given the role of CD21 and its complement ligands we wished to study their potential role with respect to BCR signaling, tyrosine phosphorylation, autoimmunity and clinical outcome in CLL. We found no evidence that autoantibodies to complement receptors and regulators caused lower expression of CD21 in CLL. Interestingly however, low CD21 expression was clearly linked with an increased CLL cell tyrosine phosphorylation potential after BCR crosslinking with sIg. Finally, lower CD21 expression was significantly associated with other markers of poor prognosis and inferior clinical outcome in CLL.

## RESULTS

**Figure 1:**
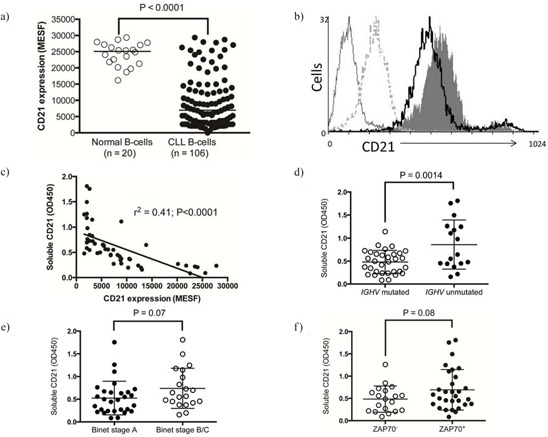
CD21 expression on normal B-cells and CLL B-cells **A.** CD21 expression was quantified on the surface of B-cells isolated from normal individuals or patients with CLL and stained for CD19, CD5 and CD21 using multi-colour flow cytometry. The MESF of CD21 expression on 5000 CD19+ cells are shown. **B.** CD21 expression measured on the surface of A20–2a3 cells after 72 hr incubation with (dashed grey line) or without mouse C3d-FC (solid grey histogram) or with FC block followed by mouse C3d-Fc (black line) or secondary antibody only (thin grey line). 5000 events were analysed, representative of 3 experiments. **C.** Categorical prognostic sub-groups of the CLL cohort were assessed for their expression of sCD21 using sandwich ELISA, **D.** CLL samples with unmutated *IGHV* genes, **E.** Binet stage B/C samples and **F.** ZAP70^+^ CLL all showed increased sCD21.

We assessed the expression level of CD21 on CLL cells isolated from 106 patients and 20 age-matched, healthy controls. The mean CD21 expression level on CLL cells was approximately 20% of that on normal B-cells which is comparable to previous reports [34–36] (Figure 1a). However, approximately 20% of CLL patients expressed CD21 levels within the normal range. In order to evaluate if C3d/immune complexes could dynamically affect CD21 expression levels on normal B-cells we exposed mouse B-cells to a C3d-Fc construct in the presence or absence of Fc blocking agents. Over a 72 h period both the Fc blocked and un-blocked cells demonstrated lower CD21 expression when exposed to C3d-Fc (Figure 1b), with the greatest effect noted with crosslinking of CD21 with Fc receptor. This data suggests that CD21 is down regulated in the presence of C3d and IgG and that crosslinking of Fc receptor to CD21 by autoantibody could result in a similar outcome.

### Soluble CD21 levels in serum are inversely correlated with surface expression of the antigen on CLL B-cells

Matched serum was available for a subset of the CLL patients in this study (*n* = 50). We therefore investigated whether surface expression of CD21 was associated with soluble CD21 in the patients’ serum. There was a strong inverse correlation between surface CD21 expression and soluble CD21 in the serum (*r*^2^ = −0.41) suggesting that some patients may be more prone to CD21 shedding (Figure 1c). Soluble CD21 was significantly higher in patients with unmutated IGHV genes (*P* = 0.0014; Figure 1d) and there was a trend towards elevated soluble CD21 in patients with advanced Binet stage disease (*P* = 0.07; Figure 1e) and those with high ZAP70 expression (*P* = 0.08; Figure 1f).

### No evidence of autoantibodies to complement regulators associated with CLL

**Figure 2:**
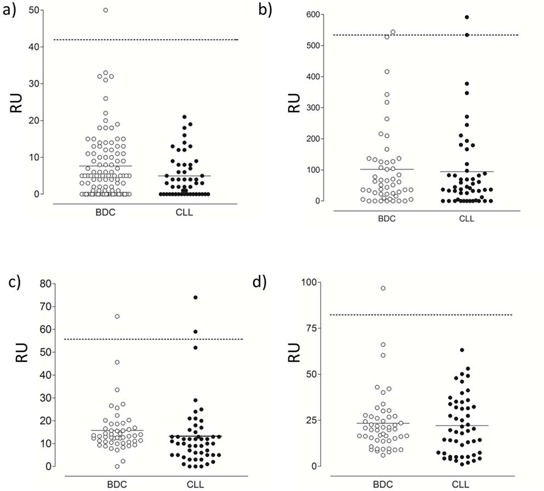
Autoantibody ELISA Serum from 50 blood donor controls (BDC) and 50 CLL patients were analysed for autoantibodies to **A.** CD21 (SCR1–2), **B.** CD35, **C.** CD46 and **D.** Factor H by ELISA. In each case, mean of subtracted data for each individual was converted to relative units (RU) using a standard curve generated using a mAb (A, C) or known positive (B, D). Each individual serum sample was measured in triplicate and the mean RU value potted. Population mean is indicated by the solid line and the 97.5 fractile of the BDC illustrated by the dashed line.

Autoantibodies to CD21 or other surface regulators could explain the lower CD21 levels commonly found on CLL cells; autoimmune phenomena are a feature of some CLL patients [37]. We hypothesized that CLL patients might produce autoantibodies to complement regulators and modify the survival of CLL B-cells. Using established ELISA protocols, we analysed 50 normal blood donor control (BDC) samples and 50 CLL samples for reactivity with recombinant proteins (CD21, CD35 and CD46) or factor H (FH) isolated from serum. Using this approach, low reactivity with all surface-bound complement regulators with the exception of CD35 was noted (Figure 2b). Both patient and control groups generated a broad range of titers, likely indicating natural blood group alloantigen differences, which were not significantly different between CLL patient and normal serum samples. In all other cases transformation of background subtracted OD450 values to relative units (RU) using a positive control antibody as a standard curve [30] confirmed that no samples reached the nominal 100 RU cut-off for positivity used as a criterion in the FH autoantibody ELISA [30]. Neither did they exceed levels measured in the normal controls using a 0.975 fractile of the BDC group to determine autoantibody positivity as recommended by the International Federation of Clinical Chemistry for data with non-normal distribution. Furthermore, western blot analysis of the highest samples confirmed non-specific reactivity (data not shown). The presence of significant autoantibodies to CD21, CD35 or CD46 in these patients is therefore unlikely.

### Low CD21 expression level correlates with other prognostic indicators

**Figure 3:**
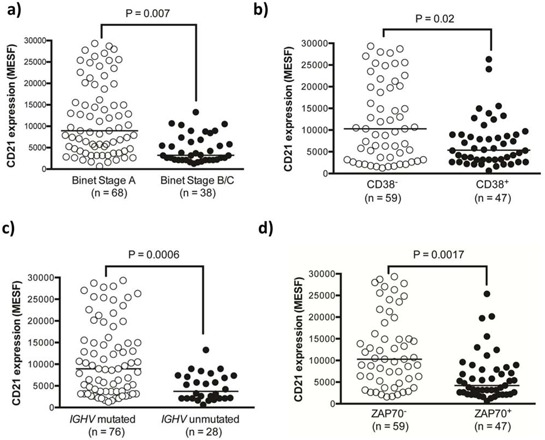
CD21 expression was significantly lower in high-risk prognostic sub-groups of CLL patients Categorical prognostic sub-groups of the CLL cohort were assessed for their expression of CD21 using multicolor flow cytometry. **A.** Binet stage B/C samples **B.** CD38^+^ CLL cells **C.** ZAP70^+^ CLL cells and **D.** CLL samples with unmutated *IGHV* genes all showed significantly reduced CD21 expression.

We next established if CD21 expression correlated with stage of disease or the other prognostic markers including CD38, ZAP70 or unmutated *IGHV*. We found that low CD21 expression level significantly correlated with all markers of poor prognosis (Figure 3a–3d). The greatest statistical correlation is with unmutated *IGHV* and ZAP70, consistent with their previously described roles in BCR signaling.

### Cells with low CD21 expression demonstrate greater p-tyrosine potential

**Figure 4:**
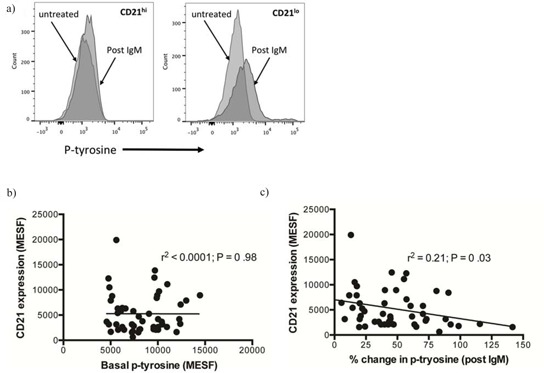
Analysis of the change in phosphotyrosine following B-cell receptor crosslinking with anti-IgM CLL cells were left untreated or treated with anti-IgM for ten minutes. They were then fixed and stained with a PE conjugated anti-phosphotyrosine antibody (PY20) and fluorescence was measured by flow cytometry. **A.** CD21^hi^ samples showed significantly less response to anti-IgM than CD21^lo^ samples. **B.** PY20 MESF and CD21 MESF levels were plotted for each patient at baseline and the data illustrates basal phosphotyrosine does not correlate with CD21 expression. **C.** The change in phosphotyrosine was plotted against CD21 expression as continuous variables and linear regression analysis was performed. There was a negative correlation between the variables suggesting that high CD21 expression on CLL cells was associated with a blunted response to B-cell receptor crosslinking with anti-IgM.

CD38^+^ CLL cells have been shown to display an increased p-tyrosine (p-TYR) response following BCR crosslinking [9]. Thus, we examined if lower CD21 expression levels also correlated with this phenomenon. Our analysis demonstrated that BCR crosslinking on CLL cells with lower CD21 expression yielded significantly greater p-TYR response than if the BCR was cross linked on CLL cells with normal levels of CD21 (Figure 4a, 4b).

### Low CD21 expression on CLL B-cells identifies a subset of patients with shorter overall survival

**Figure 5:**
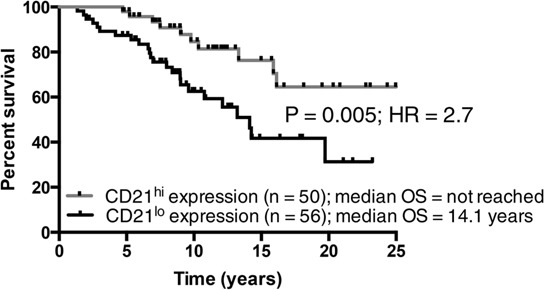
The prognostic impact of CD21 expression in CLL **A.** The median expression of CD21 was determined and the cohort was categorized into CD21^lo^ and CD21^hi^ subsets. CLL patients with a CD21^hi^ phenotype had a significantly inferior outcome when compared to CD21^lo^ patients.

Based on the data above it appeared that CD21 was a marker of progressive CLL. Analysis of survival was thus plotted using CD21 as a categorical marker using median expression to segregate the patients. Patients with ≤ median levels of CD21 expression had a much poorer prognosis than those with > median CD21 expression (Figure 5). Only 30% of patients with low CD21 were alive at 20 years post diagnosis (median survival was 14.7 years) compared to nearly 65% survival for patients with higher levels of CD21 (median survival > 25 years). These data indicate that low CD21 expression on CLL B-cells is another readily assessable marker of disease progression and prognosis.

## DISCUSSION

Given the recent data outlining the importance of the BCR in the pathogenesis of CLL we wished to explore the potential role of the known BCR co-receptor CD21 and its complement regulatory components. Consistent with earlier studies we found that CD21 expression was significantly lower in CLL cells which contradicts a smaller study showing no significant difference [38]. Furthermore, we found a strong inverse correlation between surface CD21 expression on the malignant B-cells and soluble CD21 found in the patients’ serum, similar to that noted in another recent study [39]. Of note, 10 patients analyzed herein had an 11q deletion and CLL B-cells expressing low levels of CD21. Therefore, reduced CD21 expression on CLL, is likely a mixture of transcriptional modifications [34], deletion of 11q [40], and increased activity of matrix metalloproteases [41], known to occur in CLL [42] and other disease scenarios such as RA/SLE [43]. We can speculate that the increased soluble CD21 and soluble CD23 seen in CLL is likely a consequence of a positive feedback mechanism where soluble CD21 stimulates monocyte activation via CD23. This may result in increased MMP activity which, given the described role of soluble CD23, could cleave both CD21 and CD23 from CLL cells [44, 45]. DotScan technology has been used to establish differential arrays of CD markers associated with CLL progression [46], but these do not include CD21. Our study underlines that absolute cell surface expression level of signaling molecules as compared to their presence or absence on CLL may be of equal importance.

We clearly demonstrate that lower CD21 expression had a functional consequence, as it was associated with greater BCR signaling as evidenced by tyrosine phosphorylation. We clearly demonstrated an association between lower expression levels of CD21 on CLL cells and poorer clinical prognosis. Low CD21 expression and other markers of poor prognosis such as advanced Binet stage, CD38^+^, ZAP70^+^ and unmutated *IGHV* correlated with a worse clinical outcome. Thus, low CD21 expression on CLL cells represents an additional prognostic biomarker intimately involved in BCR signaling.

CD38^+^ CLL cells are reported to express higher levels of ZAP70 [47], a molecule known to enhance BCR signaling [8]. In light of our data, we might speculate that CD38, ZAP70 and CD21 are part of a common signalosome, which has arisen as part of disease progression. The strongest association noted in this study was between low CD21 expression and unmutated *IGHV* genes. This supports the hypothesis that the array of changes observed in CLL cells is linked to reactivity to self-antigen. It is also notable that the cellular features of CLL cells with mutated *IGHV* genes (largely unresponsive to anti-IgM) are similar to B-cells that have undergone receptor desensitization following chronic antigen stimulation [48].

Furthermore, there is now increasing interest in CD21^lo (/negative)^ B-cells in association with autoimmune conditions [49, 50] and there may be common mechanism(s) driving the generation of these B-cells in both disease settings. CLL patients have an increased risk of the development of autoimmunity, including autoimmune hemolytic anemia, Evan's syndrome, idiopathic thrombocytopenia purpura (ITP), Hashimoto's thyroiditis, vasculitis and rheumatoid arthritis [37]. Could this be linked to the low CD21 expression? Certainly, CD21 expression level is directly linked to self-tolerance [51] and reduced expression levels have been suggested as a risk factor in developing autoimmunity [52]. Increased C3d production during inflammation may lead to reduced CD21 surface expression [53] and this is supported by our data with C3d-Fc and chronic CD21 signaling [54]. A further consideration was that autoantibodies to CD21, as seen in rheumatoid arthritis [55], could provide survival signals for autoreactive B-cells and potentiate disease. Our analysis herein establishes that no antibodies directed against CD21 or CD35, or key complement regulators exist that could mediate such survival signals. Furthermore, although our *in vitro* data indicates that CD21 levels can be markedly down-regulated on exposure to C3d-Fc immune-complexes, no overt signaling changes were noted. This suggests that this aspect may not be causal for the changes observed in CLL.

In conclusion, our data indicate that low CD21 expression on CLL cells is intrinsically a marker of poor prognosis in the disease and support the growing evidence that BCR cell signaling pathways play a key role in modulating the clinical course of this disease. It remains unclear, whether altered CD21 expression contributes to malignancy or is a consequence of the disease. To date, investigations relating to the ability of CLL cells to signal have focused on ligation of the BCR using anti-IgM and IgD antibodies [9]. Although differential signaling between CLL cells and controls was observed, they did not relate to altered IgM, CD20 or CD79b expression levels. The present study identifies loss of CD21 as a potential factor in modulation of these signals and that further studies are required to delineate the integral roles of CD21, CD38, ZAP70 and *IGHV* mutation status in BCR signaling and how they may alter the responsiveness of CLL cells to small molecule inhibitors

## MATERIALS AND METHODS

### Patient recruitment

For ELISA analysis, serum was donated by 100 normal healthy blood donors (blood donor controls, BDC; normally distributed, ranging from 17 to 72 years of age, median age 46, 54% female, 98% White-Caucasian) via the blood transfusion service (NHSBT, Newcastle upon Tyne). Peripheral blood was obtained from 106 serially collected CLL patients (see Table 1 for clinical profile) and 20 age-matched normal controls following informed, written consent in accordance with the Declaration of Helsinki and with local ethical committee approval (02/4806). Patient samples were collected based on a confirmed diagnosis of CLL.

**Table 1:** Clinical characteristics of the CLL discovery cohort (*n* = 106)

Factor	Subset	Number
Median Age		66 years
Range		32 – 91 years
Median Follow-up		9.6 years
Required treatment	Treated	45
	Untreated	61
Binet stage	A	68
	B/C	38
CD38 expression	<20%	59
	≥20%	47
Genetics	11q- / 17p-	13
	N / O	68
	Not Determined	25
*IGHV* Status	<98%	76
	≥98%	28
	Not Determined	2
ZAP-70 expression	<20%	59
	≥20%	47

11q- and 17p-: any FISH or karyotypic abnormality of 11q or 17p

N: No detectable cytogenetic aberration by fluorescence *in situ* hybridization (FISH)

O: Other cytogenetic abnormality (excluding 11q- or 17p-)

*IGHV* status: <98% sequence homology with the closest germline sequence (mutated); ≥98% sequence homology with the closest germline sequence (unmutated)

### Antibodies

Purified GB24 (mouse monoclonal raised against human MCP (CD46) kindly gifted by Prof John Atkinson/Dr Paula Bertram (School of Medicine, Washington University, St. Louis, MO, USA). Purified 171 (BD Pharmingen, Oxford, UK) Knops Antigen, Kn^a^ (International blood group reference laboratories, NHS BTS, Bristol, UK). Goat anti-human IgG specific-HRPO and sheep anti-mouse IgG HRPO were obtained from Stratech Scientific (UK)

### ELISA

#### Autoantibody ELISA

The autoantibody ELISAs were carried out as previously described [30, 31]. In brief, 96-well plates were coated with 2–5 μg/ml of recombinant (r)CD21 (scr1–2), rCD35, rCD46 or purified factor H in phosphate buffer saline (PBS) and incubated overnight at 4°C. A PBS only (no coat) plate was also included. Plates were blocked with PBS, 0.1% Tween 20 (PBST) and sera, diluted 1/50 in PBST, was added in triplicate wells for 1 hour at room temperature. The plates were washed with PBST and bound antibody detected with goat anti-human IgG-HRPO (1/20,000). Tetramethylbenzidine (TMB; AbDserotec) was used to develop the assay. The absorbance at 450nm was read (SpectraMax 190; MDS Analytical Technologies, Ltd., Coventry, UK). Readings from the PBS only plate were subtracted from the antigen plate data and means calculated for triplicates. Monoclonal antibodies (171 and GB24 for CD21 and CD46, respectively) and known positive antisera (for CR1 and factor H, Knops antigen positive and ‘French’ positive, respectively [30, 31]) were included as positive controls. Pooled normal healthy donor serum and secondary antibody only (goat anti-mouse and goat anti-human IgG HRPO only) served as negative controls.

#### Detection of sCD21

All steps were carried out at room temperature for 1 hour unless stated. Nunc Maxisorb^™^ plates were coated with 10 μg/ml of monoclonal antibody 171 [32] in pH 9.6 carbonate buffer and incubated overnight. Plates were blocked with PBS/1% bovine serum albumin (BSA). Sera, diluted 1/50 in PBS/BSA, were added in triplicate. The plates were washed with PBS 0.01% Tween20 (4 times) and bound antibody detected with an affinity purified sheep anti-human CD21 (1/2000; AF4909, R&D systems, UK) diluted in PBS/BSA. After washing, bound antibody was detected using a donkey anti-goat-HRPO (1/5000; Stratech Scientific ltd, UK) antibody. Tetramethylbenzidine (TMB; AbDserotec) was used to develop the assay as above. Average of ‘no serum/background’ wells was subtracted from all samples and the average of each triplicate for each sample is shown as an adjusted OD 450 value.

### Flow cytometry

#### CD21, CD38 and ZAP-70 expression

Cell surface expression of CD21 and CD38 were examined by four-color flow cytometry. In brief, CLL cells were labeled with CD5-FITC, CD38-PE, CD21-PERCP/cy5.5 and CD19-APC. CD21 expression was recorded for each sample as mean fluorescence intensity (MFI) values and subsequently converted to molecules of equivalent soluble fluorochrome (MESF) in order to standardize the data and control for day-to-day variation in the Accuri C6 cytometer performance (Becton Dickinson). A calibration curve was constructed by monitoring a mixture of fluorospheres (Dako) labeled with known amounts of fluorochrome. For categorical analysis, </≥ the median MESF value for CD21 was used to divide the cohort. The cut-off point used for CD38 positivity in the CLL population was ≥20%. Cytoplasmic ZAP70 expression was also determined by flow cytometry as described previously and the cut-off point for positivity was ≥20% [33]. The expression of CD21 on normal B-cells was determined by gating CD19^+^ B-cells.

#### Intracellular expression of tyrosine phosphorylated proteins

CLL lymphocytes were analyzed by three-color immunofluorescent staining using CD5 and CD19 surface antigenic markers in conjunction with a PE-labeled phosphorylated-tyrosine antibody (PY20). Changes in phosphorylated tyrosine were monitored before and after BCR cross-linking. Briefly, BCR ligation was achieved by the addition of 20 μg/ml goat anti-IgM (Southern Biotechnology Associates) for 15 mins at 37°C. Subsequently the cells were labeled with CD5-PE and CD19-APC and then fixed and permeabilized using a Fix and Perm kit according to the manufacturer's instructions (Caltag). The cells were then labeled with a FITC-phosphorylated-tyrosine (PY20) antibody (Santa Cruz Biotech) and analyzed on an Accuri C6 flow cytometer. At least 10,000 events were acquired from each sample and the mean recorded. The MFI values for phosphorylated-tyrosine were converted to MESF values using fluorosphere-derived calibration curve as described above.

### IGHV mutational analysis

Amplification and analysis of immunoglobulin *(IGHV)* gene mutation status was assessed using BIOMED II primers as described previously [32]. *IGHV* sequences were compared with the IGMT database (http://imgt.cines.fr) and samples with ≥98% homology to germline sequences were considered unmutated.

### Statistical analysis

Statistical analysis was carried out using Prism 6.0 (Graphpad) and SAS version 9.3 software (SAS Institute). Normal distribution of the ELISA data was established by skewness, kurtosis and the Shapiro-Wilks tests. Univariate comparisons were made using the Student's *t*-test for paired and unpaired observations. The correlation between CD21 expression and the change in phosphotyrosine (MFI) was performed using least squares linear regression. All data was confirmed as Gaussian or a Gaussian approximation using the omnibus K2 test. Overall survival (OS) for categorical sub-groups was assessed with the log-rank test and displayed as Kaplan-Meier curves.
